# Aberrant Phase Separation of FUS Leads to Lysosome Sequestering and Acidification

**DOI:** 10.3389/fcell.2021.716919

**Published:** 2021-10-22

**Authors:** Franziska Trnka, Christian Hoffmann, Han Wang, Roberto Sansevrino, Branislava Rankovic, Benjamin R. Rost, Dietmar Schmitz, H. Broder Schmidt, Dragomir Milovanovic

**Affiliations:** ^1^Laboratory of Molecular Neuroscience, German Center for Neurodegenerative Diseases (DZNE), Berlin, Germany; ^2^Laboratory of Network Dysfunction, German Center for Neurodegenerative Diseases (DZNE), Berlin, Germany; ^3^Berlin Institute of Health, NeuroCure Cluster of Excellence, Charité—Universitätsmedizin Berlin, Corporate Member of Freie Universität Berlin and Humboldt-Universität zu Berlin, Berlin, Germany; ^4^Department of Biochemistry, Stanford School of Medicine, Stanford, CA, United States

**Keywords:** phase separation, lysosomes, ALS, optodroplets, FUS, lyso-pHluorin

## Abstract

Amyotrophic lateral sclerosis (ALS) is a progressive neurodegenerative disease that leads to the death of upper and lower motor neurons. While most cases of ALS are sporadic, some of the familial forms of the disease are caused by mutations in the gene encoding for the RNA-binding protein FUS. Under physiological conditions, FUS readily phase separates into liquid-like droplets *in vivo* and *in vitro*. ALS-associated mutations interfere with this process and often result in solid-like aggregates rather than fluid condensates. Yet, whether cells recognize and triage aberrant condensates remains poorly understood, posing a major barrier to the development of novel ALS treatments. Using a combination of ALS-associated FUS mutations, optogenetic manipulation of FUS condensation, chemically induced stress, and pH-sensitive reporters of organelle acidity, we systematically characterized the cause-effect relationship between the material state of FUS condensates and the sequestering of lysosomes. From our data, we can derive three conclusions. First, regardless of whether we use wild-type or mutant FUS, expression levels (i.e., high concentrations) play a dominant role in determining the fraction of cells having soluble or aggregated FUS. Second, chemically induced FUS aggregates recruit LAMP1-positive structures. Third, mature, acidic lysosomes accumulate only at FUS aggregates but not at liquid-condensates. Together, our data suggest that lysosome-degradation machinery actively distinguishes between fluid and solid condensates. Unraveling these aberrant interactions and testing strategies to manipulate the autophagosome-lysosome axis provides valuable clues for disease intervention.

## Introduction

Amyotrophic lateral sclerosis (ALS) is a devastating neurodegenerative disease leading to the degeneration and death of motor neurons in the spinal cord, brainstem and motor cortex (Kiernan et al., 2011; Brown and Al-Chalabi, 2017). This results in progressive paralysis, causing the majority of patients to die within several years upon diagnosis (Taylor et al., 2016). While most cases of ALS are sporadic, about 10% are familial forms caused by mutations in different genes (Hardiman et al., 2017), including those encoding the cytoplasmic enzyme superoxide-dismutase 1 (Rosen et al., 1993), the RNA-binding proteins TAR DNA-binding protein 43 (Kabashi et al., 2008) and fused in sarcoma/translocated in liposarcoma (FUS) (Kwiatkowski et al., 2009; Vance et al., 2009). ALS-linked mutations in these proteins often lead to the formation of intracellular protein aggregates in both neuronal and glial cells (Blokhuis et al., 2013). Genetic studies indicate that FUS is mutated in ∼5% of familial forms and ∼1% of sporadic cases of ALS (Kiernan et al., 2011). Pathological examinations of post-mortem tissues from patients with FUS mutations show predominant degeneration of lower motor neurons, with large cytoplasmic aggregates positively stained for FUS in motor neurons and surrounding glia (Bäumer et al., 2010; Blokhuis et al., 2013). Two hotspots for ALS-linked mutations in FUS are within its nuclear localization sequence, resulting in the accumulation of FUS in the cytoplasm (Vance et al., 2013), and the N-terminal intrinsically disordered region (IDR), promoting aggregation (Murakami et al., 2015; Patel et al., 2015).

Notably, the presence of IDRs is a common feature of many proteins mutated in familial forms of ALS (e.g., TDP43, FUS, hnRNPA1) (Li et al., 2013). In the last years, proteins with IDRs were shown to readily phase separate into liquid-like droplets and hydrogels, both in cells and *in vitro* (Kim et al., 2013; Molliex et al., 2015). Liquid-liquid phase separation is a process during which one or multiple components in the same state segregate from another component into distinct compartments, for example, demixing of oil in water. In the context of cell biology, it is a process where (bio)polymers separate out from homogenous aqueous mixtures into dynamic assemblies referred to as biomolecular condensates (Banani et al., 2017; Shin and Brangwynne, 2017). ALS-linked mutations in the IDR of FUS often promote aberrant liquid-to-solid transitions (Patel et al., 2015; Niaki et al., 2020), suggesting that they are also crucial drivers of aggregation in disease (Blokhuis et al., 2013).

Intriguingly, recent work has shown that biomolecular condensates can both interact with and dynamically incorporate membrane-bound organelles. For example, phase separation of synapsin 1 and α-synuclein, two major synaptic proteins with IDRs, results in the clustering of synaptic vesicles (Milovanovic et al., 2018; Hoffmann et al., 2021). Similarly, membrane organelles such as ER affect the dynamics of ribonucleoprotein particles and stress granules (Liao et al., 2019; Lee et al., 2020). These emerging links between condensates and membrane-bound organelles are poised to play an important role both in physiology and diseases especially through the cellular quality control pathways such as the autophagy-lysosome system (Wallings et al., 2019). However, the link between cytosolic protein/RNA condensation, protein aggregation and organelle dynamics remains elusive.

Here, we systematically analyze the effect of FUS condensation—triggered by high concentration, mutations, and chemical stress—on the dynamics of lysosomes. We show that aggregates of FUS, but not fluid condensates, result in the accumulation of lysosomes. This cellular response is independent of whether we induce FUS aggregation by overexpression or mutagenesis. In fact, our data indicate that the cytosolic concentration of FUS has a dominant effect on its aggregation over the mutations. We further employ optically-induced FUS condensation (Shin et al., 2017) using a Cry2-oligomerization system and genetically encoded reporters of lysosome acidity (Rost et al., 2015, 2017) to show that functional lysosomes accumulate around the aberrant FUS condensates.

## Materials and Methods

### Cloning

The pCry2-mCh-MCS plasmid was prepared by Gibson assembly and cloned in the pmCherry-C1 backbone. The expression cassette was under the CMV promoter and SV40 terminator (for detailed sequence, see Supplementary Table 1). The DNA fragment encoding the IDR of human FUS (residues 1-215, extracted from Addgene plasmid #60362) was inserted by PCR-based cloning into the pCry2-mCh vector using *Xho*I-*Kpn*I restriction sites (Supplementary Table 2). To introduce changes (exchange or deletion) in DNA sequence of abovementioned plasmids, pCry2-mCh-FUS and FUS-EGFP, we performed PCR mutagenesis. Primers were designed in an overlapping fashion covering the region of interest: 10 bp upstream and at least 17 bp downstream of the region are complementary to the plasmid template (Supplementary Table 2). LAMP1-mScarlet was prepared by restriction enzyme-based cloning from LAMP1-RFP (kind gift from Geert van den Bogaart) using *Nhe*I-*Bam*HI into pmScarlet-I N1.

### Cell Culture and Transfection

Human embryonic kidney (HEK 293) cells were cultured in a humidified incubator at 37°C with 5% CO_2_ using Dulbecco’s modified eagle medium (DMEM) containing 4.5 g/L D-Glucose (Gibco) supplemented with 10% heat-inactivated FBS, 1% Penicillin/Streptavidin and 1% MEM medium (non-essential amino acids) (Sigma). When reaching a confluency of 80–90%, cells were passaged using standard trypsinization techniques. For live-cell imaging experiments, HEK cells were either grown on glass coverslips (25 mm) or in glass bottom dishes until reaching a confluency of 50–70%.

Cells were transfected by Lipofectamine 2000 (Thermo Fisher Scientific) following manufacturer’s instructions. Briefly, 3 μl of lipofectamine 2000 was mixed with 1 μg of each construct (unless indicated otherwise in the figure legends) in 200 μl OptiMEM (Gibco) for HEK cells. When co-transfected 2 μg of total DNA was used in equimolar ratio. Transfection mix was incubated for 30 min at room temperature, then was added to the cells. Cells were transfected and incubated overnight (37°C and 5% CO_2_). The day after medium was fully replaced with fresh supplemented DMEM. Images were acquired 20 h after transfection.

### Western Blot for Analysis of FUS-Protein Expression Level

Transfected HEK 293 cells expressing FUS-EGFP variants were collected and washed with 1 mL ice-cold 1 × PBS, centrifuged and resuspended in RIPA buffer (Sigma R0278, supplemented with protease inhibitors Complete EDTA-free from Roche) at a density of 1 × 10^7^ cells/mL. After 30 min on ice, lysates were cleared by centrifugation for 15 min at 20,000 × g at 4°C. Total soluble protein concentration was determined by BCA assay (Pierce 23227). For Western blot, 25 μg of total soluble protein were analyzed on a 12% Tris-Glycine SDS-PAGE gel and transferred onto Ambersham HybondLFP membrane (0.2 μm, Cytiva). Wet transfer was done overnight at 10 V at 4°C. Blocking with 5% Difco skim milk (MLK) in TBS was performed for 1 h at room temperature and primary antibody incubation overnight with slow agitation at 4°C: anti-GFP (1:4,000 in 5% MLK/TBS, Abcam ab290), anti-α-tubulin (1:2,000 in 5% MLK/TBS, Sigma T6199). For fluorescent detection Cy5- or Cy3-conjugated secondary antibodies (goat-anti-rabbit-IgG-Cy5, goat-anti-mouse-IgG-Cy3) from the Amersham ECL Plex^TM^Detection kit were used according to manufacturer’s instructions (1:2,500 in 5% MLK/TBS, Cytiva). Fluorescence detection is done using the Typhoon TrioVariable Imager System (GE Healthcare).

### Microscopy

Live cell imaging was performed on a Nikon spinning disk confocal CSU-X microscope equipped with a temperature stage at 37°C and a 5% CO_2_ saturation. A planar Apo objective 60 × 1.49- NA was used. Excitation wave lengths were: 488 nm for EGFP; 561 nm for mCherry. Image Analysis was done using Fiji (ImageJ Version: 1.8.0_172/1.53c) software.

### Formation of Condensates by Photoactivation

For photoactivation (optogenetic control), cells expressing Cry2-mCherry-FUS variants were imaged by use of two laser wavelengths (488 nm for Cry2 activation, 561 nm for mCherry imaging), similarly as in Shin et al. (2017). Applied laser intensities at the output were 0.01, 0.02, 0.04, and 0.15 mW corresponding to 1, 3, 5, and 10% laser power, respectively. Activation times for photoactivation are indicated in the respective figure legends.

### Enrichment of FUS in Condensates After Photoactivation

HEK cells expressing Cry2-mCherry-FUS variants were activated for either 1 or 10 min at 0.15 mW 488 nm-laser power. Z-stack images of activated cells are collected immediately upon completion of photoactivation and 30 min after the activation laser was turned off. The cytosolic signal intensity is compared between these two time points and plotted as box-whisker plots to indicate fold change of cytosolic fluorescence intensity.

### Condensate Dispersion Assay With 1,6-Hexanediol

HEK cells expressing Cry2-mCherry-FUS variants were seeded on glass bottom dishes and photoactivation was performed to obtain condensate formation as indicated. 1,6-Hexanediol was added to the medium (3% final concentration), and the images were acquired after 4 min of incubation.

### Cell Stress Induction

To analyze the effect of stressed cells on the architecture of aggregates and the arrangements of lysosomes during live-cell-imaging, media of HEK cells was replaced with 0.5 mM sodium arsenite in DMEM medium containing 4.5 g/L D-Glucose (Gibco) supplemented with 10% heat-inactivated FBS, 1% Penicillin/Streptavidin and 1% MEM medium (non-essential amino acids) (Sigma). Images were obtained before and after 60 min of treatment.

### Visualization of Lysosome Acidification

To assess the acidity of lysosomes, lyso-pHluorin (CMV-pHluorin-CD63-SV40) was transfected together with desired constructs in HEK cells. To increase the pH during live-cell-imaging, ammonium chloride was added to the media (50 mM final concentration). Images of the same region of interest were obtained directly before and after treatment. To determine the overlapping surface between lysosomes (lyso-pHluorin) and FUS condensates (Cry2-FUS), we used the IMARIS software (Oxford Instruments). The volumes were determined for each channel based on the acquired z-stacks of cells, followed by the calculation of signal colocalization. The fraction of juxtaposed lysosomes was calculated as the volume of overlapping channels divided by the total volume of FUS fluorescence. For each of FUS constructs (wild-type, G156E, ΔFibril), we used cells from three independent transfections. Obtained fractions of juxtaposed lysosomes to each of the FUS constructs were compared with the two-sided unpaired *t*-test.

## Results

### Manipulation of Cry2-FUS Constructs by Photoactivation Allows the Formation of Reversible Condensates and Insoluble Aggregates

**FIGURE 1:**
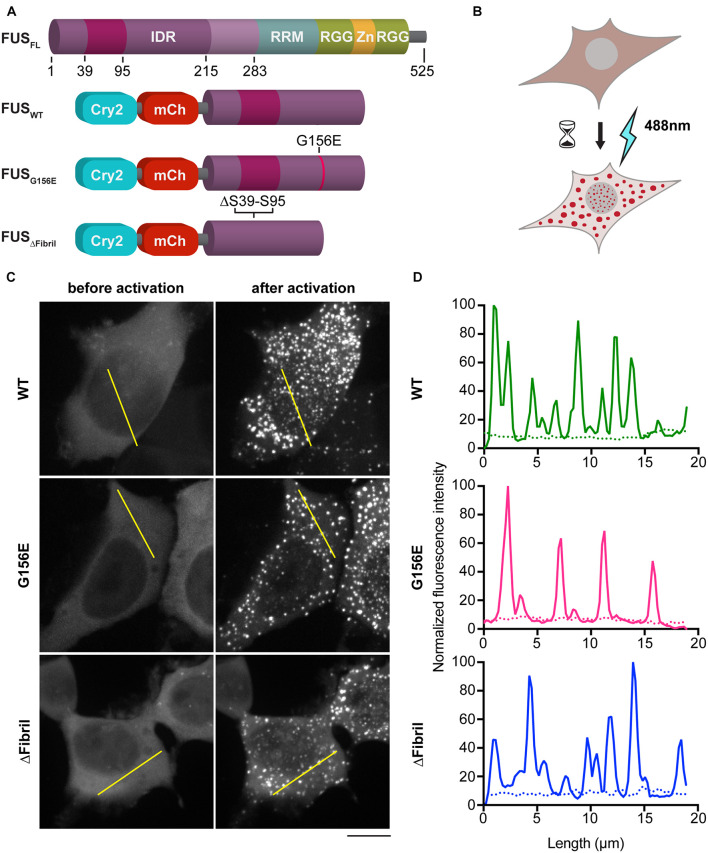
Photoactivation of FUS condensation. **(A)** Scheme of full-length FUS protein (top) and chimeric constructs used in this study (bottom) (IDR, intrinsically disordered region; RRM and RGG, RNA-binding motives; Zn, Zn-finger). **(B)** Cartoon of the imaging assay in HEK cells. **(C)** Representative images (z-projections) before (left) and after (right) photoactivation of 1 min at 0.02 mW 488 nm-laser power at the output. Scale bar, 10 μm. **(D)** Line profiles of highlighted regions in **(C)** (dotted line, before activation; full line, after activation).

FUS contains an N-terminal IDR and forms dynamic compartments with liquid-like properties both *in vitro* and *in vivo* (Figure 1A; Burke et al., 2015; Patel et al., 2015; Murthy et al., 2019). ALS-causing mutations such as G156E are known to accelerate the conversion of FUS liquid condensates into insoluble aggregates (Patel et al., 2015). Indeed, IDR-containing proteins, including FUS, can mature over time into hydrogels or amyloid-like fibrils (Kato et al., 2012; Lin et al., 2015; Nott et al., 2015; Wei et al., 2017). Moreover, recent studies revealed the presence of a region within residues 30–95 of FUS that is prone to engage in amyloid-like interactions that structurally explain the aging of liquid FUS condensates into solid aggregates (Murray et al., 2017). Yet, how cells sense the material properties of their phase separated compartments and triage aberrant condensates remains poorly understood. To shed light on this, we employed a photoactivation system based on the plant flavoprotein, cryptochrome 2 (Cry2), which contains a light-sensitive domain that forms oligomeric photobodies in response to blue light (Bugaj et al., 2013). Specifically, we fused Cry2 to the N terminal IDR of FUS to generate the so-called optodroplets (Figures 1A,B; Shin et al., 2017).

**FIGURE 2:**
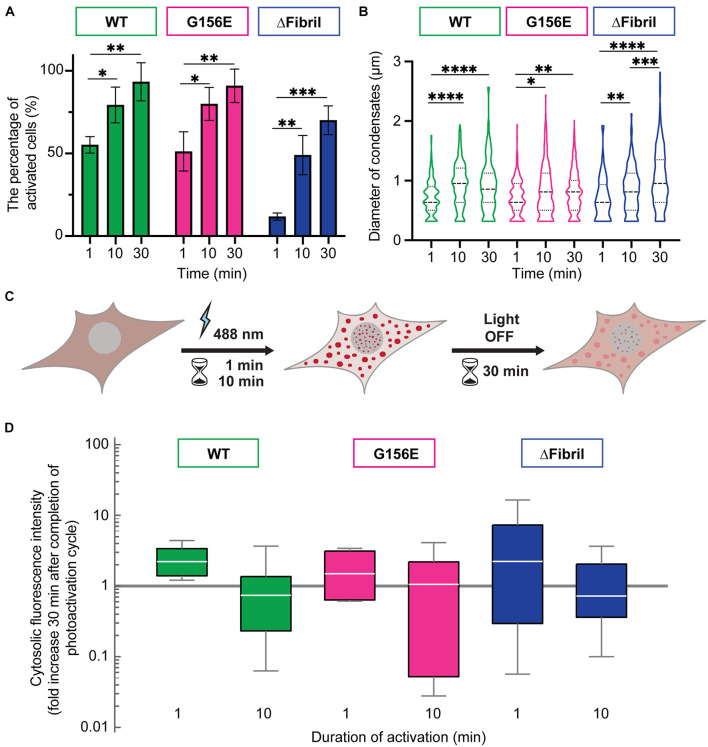
Longer photoactivation promotes FUS condensation. **(A)** The percentage of activated cells after 1, 10, or 30 min of activation (0.15 mW 488 nm-laser power) for WT (green), G156E (magenta) and ΔFibril (blue) FUS. **(B)** Same as **(A)**, but now showing the diameter of condensates (dashed line—median value; dotted line—interquartile range). **(C)** Scheme of the imaging-based assay for determining the enrichment of FUS in condensates after completion of photoactivation. Cytosolic signal intensity is compared immediately after photoactivation with the intensity 30 min after the activation laser is turned off. **(D)** Box-Whisker-Plots showing fold change of cytosolic fluorescence intensity 30 min after photoactivation (1 or 10 min) at 0.15 mW 488 nm-laser power for WT (green), G156E (magenta) and ΔFibril (blue) FUS. Bars represent average values with standard deviation. ^∗^*P* < 0.05, ^∗∗^*P* < 0.01, ^∗∗∗^*P* < 0.001, ^****^P < 0.0001 two-sided, unpaired *t*-test; each experiment is done in three different rounds of transfections.

In addition to the wild-type (WT) FUS optodroplet construct, we also generated G156E point mutant and fibril core deletion (a.a. deletion 39–95; ΔFibril) variant to examine their ability to form condensates. Our data indicate that all three constructs were enriched in puncta upon photoactivation (Figures 1C,D). These puncta had a similar diameter (median 0.6 μm) for WT, G156 and ΔFibril FUS (Supplementary Figure 1A). The condensation is significantly lower for FUS ΔFibril, where only a fraction of transfected cells showed puncta upon photoactivation (Supplementary Figure 1B). The puncta formation was sensitive to the laser intensity (Supplementary Figure 2) and duration of photoactivation (Figures 2A,B). Cry2 construct alone remains soluble even after 10 min of photoactivation (Supplementary Figure 6).

**FIGURE 3:**
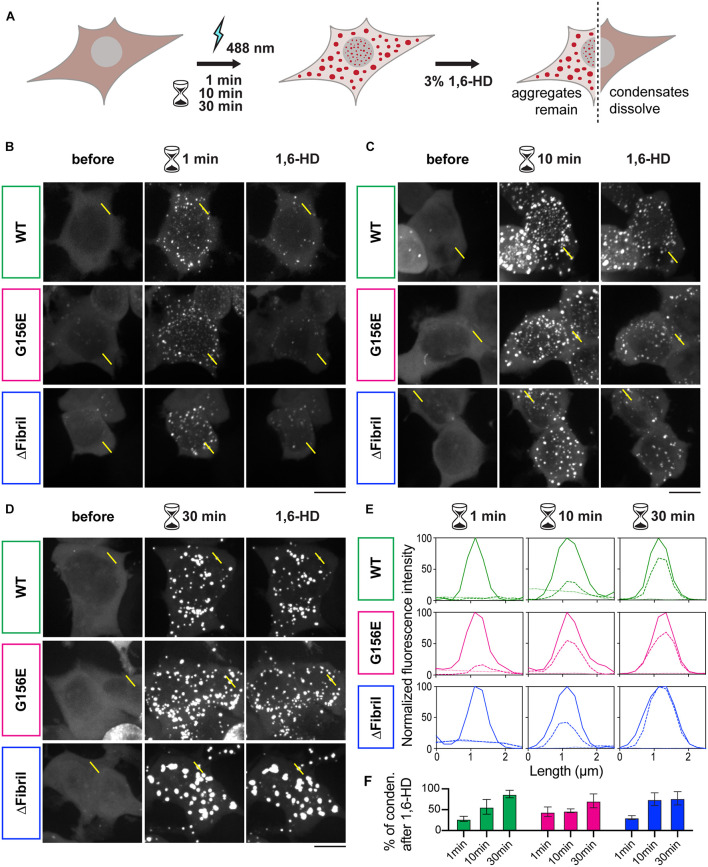
Continuous photoactivation results in the formation of solid-like FUS condensates. **(A)** Scheme of the imaging-based assay for the formation of FUS condensates coupled to 1,6-hexanediol (1,6-HD) for dissecting whether condensates have liquid- or solid-like properties. **(B)** Images (z-projections) of wild-type FUS (top), FUS G156E (middle) and ΔFibril (bottom) before, after 1 min photoactivation at 0.15 mW 488 nm-laser power at the output, and upon the addition of 3% 1,6-HD. **(C)** Same as in B, but photoactivation for 10 min. **(D)** Same as in **(B)**, but photoactivation for 30 min. Scale bars, 10 μm. **(E)** Line profiles of highlighted regions in B-D (dotted line, before activation; full line, after activation; dashed line, after 1,6-hexanediol treatment). **(F)** Fractions of condensates that remain after 1,6-hexanediol treatment (green, Cry2-FUS wild-type; magenta, Cry2-FUS G156E; blue, Cry2-FUS ΔFibril). Bars represent average values with standard deviation; each experiment is done in three independent rounds of transfection.

Next, we wanted to assess whether the puncta formed upon photoactivation of Cry2-FUS constructs were indeed condensates and at what point these condensates transition from the fluid-like structures into solid-like aggregates. For example, photoactivated Cry2-mCherry-FUS condensates remain enriched and segregated from the cytosol even 30 min after completion of the activation (Figures 2C,D). In fact, photo-oligomerization was shown to be a powerful approach to drive condensates into a supersaturation regime where liquid phases transition into hydrogels or aggregates (Shin et al., 2017; Wei et al., 2017; Bracha et al., 2018). A facile assay to dissect whether condensates are in the liquid- or solid-like state is the use of an aliphatic alcohol 1,6-hexanediol (Kroschwald et al., 2015, 2017; Schmidt and Görlich, 2015; Lin et al., 2016). To achieve this, we coupled the Cry2-FUS photoactivation with a 1,6-hexanediol perfusion system that allowed us to dissect the fluid nature of condensates (Figure 3A). For each of the Cry2-FUS constructs, we analyzed the reversibility of condensates after 1 min (Figure 3B), 10 min (Figure 3C), and 30 min (Figure 3D) of photoactivation. Our data show that with the increase of the activation time, the Cry2-FUS constructs can be triggered to form insoluble aggregates resistant to 1,6-hexanediol treatment irrespective of whether we analyze the wild-type or mutated sequence (Figures 3E,F).

### Local Concentration of FUS in the Cytosol Determines Its Condensation State

**FIGURE 4:**
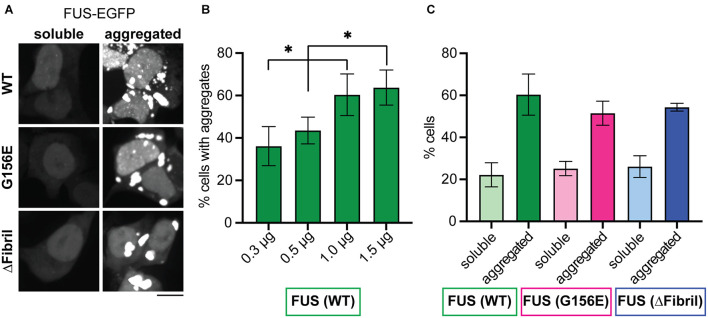
Increasing concentration of cytosolic FUS leads to aggregation for both wild-type and mutated proteins. **(A)** Representative images (z-projections) of HEK cells expressing full-length FUS-EGFP (wild-type, top; G156E middle, ΔFibril, bottom) show both diffuse signal and aggregates. Scale bar, 10 μm. **(B)** The percentage of cells that contain aggregates of FUS with the increased amounts of transfected FUS-EGFP construct. **(C)** The percentage of cells that contain either soluble or aggregated wild-type FUS (green), FUS G156E (magenta), and FUS ΔFibril (blue) upon transfection with 1 μg of the corresponding construct. Bars represent average values with standard deviation; ^∗^*P* < 0.05, two-sided, unpaired *t*-test; each experiment is done in three different rounds of transfections with at least two hundred analyzed cells for each condition.

As continuous photoactivation promotes the enrichment of Cry2-containing FUS constructs into condensates and drives their aging, we sought to further analyze the effect of the total FUS concentration on its condensation state. To this end, we transfected an increasing amount of plasmids containing full-length FUS-EGFP constructs for WT, G156E or ΔFibril variants. All constructs are expressed at a similar level (Supplementary Figure 3A). Cells were referred to as soluble when the FUS-EGFP constructs gave diffuse signal or aggregated when FUS-EGFP constructs formed large, irregular-shaped inclusions (Figure 4A and Supplementary Figure 3B). In agreement with the photoactivation of Cry2-FUS, the expression of increasing amounts of plasmid encoding for the full-length FUS-EGFP results in an increase of observed aggregates (Figure 4B and Supplementary Figure 4A). In fact, tripling the amount of transfected plasmid resulted in a significant increase of cells that form cytoplasmic aggregates. Accordingly, the fraction of cells with soluble signal dropped with the increase of transfected plasmid (Supplementary Figure 4B). Interestingly, a similar effect of the accumulation of FUS-EGFP aggregates was detected for FUS G156E and FUS ΔFibril (Figure 4C and Supplementary Figure 4C). These data imply that the total intracellular concentration of FUS has a dominant effect on its condensation, aligned with the concept that concentration plays an essential role in determining the phase behavior (Shin and Brangwynne, 2017; Wei et al., 2017).

### Aggregates but Not Liquid Condensates of FUS Sequester Mature Acidic Lysosomes

**FIGURE 5:**
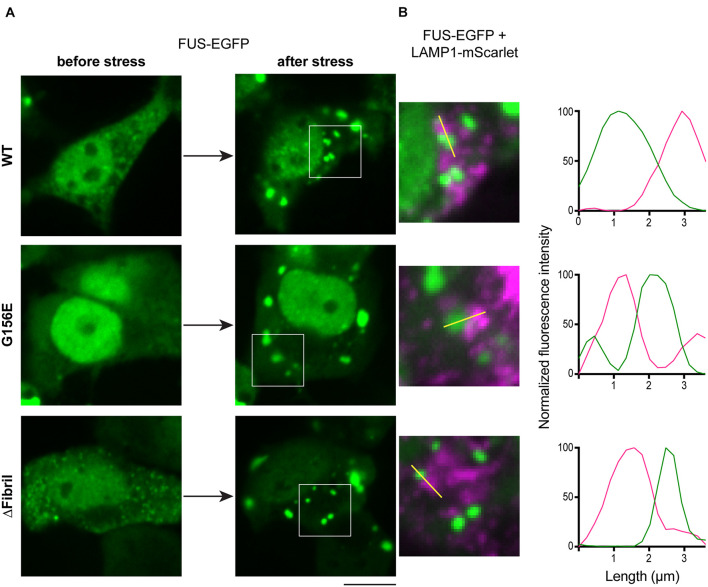
Stress induces FUS aggregation and lysosome accumulation. **(A)** Cells transfected with FUS-EGFP (top), FUS-EGFP G156E (middle), and FUS-EGFP ΔFibril (bottom) imaged before (left) and after (right) incubation with sodium arsenite for 60 min. Scale bar, 10 μm. **(B)** Accumulation of lysosomes (LAMP1-mScarlet) juxtaposed to FUS aggregates. Left: magnified regions from images in **(A)**; right: Line profiles of the highlighted region indicated with yellow line.

FUS aggregation in response to cellular stress is one of the hallmarks in ALS pathology (Groen et al., 2013). Recent studies suggest that cells use various protein quality control mechanisms to prevent and possibly reverse the aggregation of FUS and related IDR-containing proteins (Mandrioli et al., 2019). However, whether cells recruit lysosomes to help triage aggregates remains poorly understood. To visualize whether lysosomes respond to FUS aggregation, we exposed cells to acute oxidative stress using sodium arsenite (Sama et al., 2013). Sodium arsenite-induced cellular toxicity leads to the formation of stress granules and protein aggregates (Wheeler et al., 2016), which allowed us to image the organelle response upon this acute perturbation. Specifically, we co-transfected HEK cells with FUS-EGFP constructs and lysosome-associated membrane protein 1 (LAMP1-mScarlet), a marker of the endolysosomal organelles (Figure 5A; Cheng et al., 2018a). Indeed, upon 1 h of exposure to sodium arsenite, aggregates of FUS-EGFP formed, and lysosomes (i.e., LAMP1-mScarlet signal) were juxtaposed to these aggregates (Figure 5B).

**FIGURE 6:**
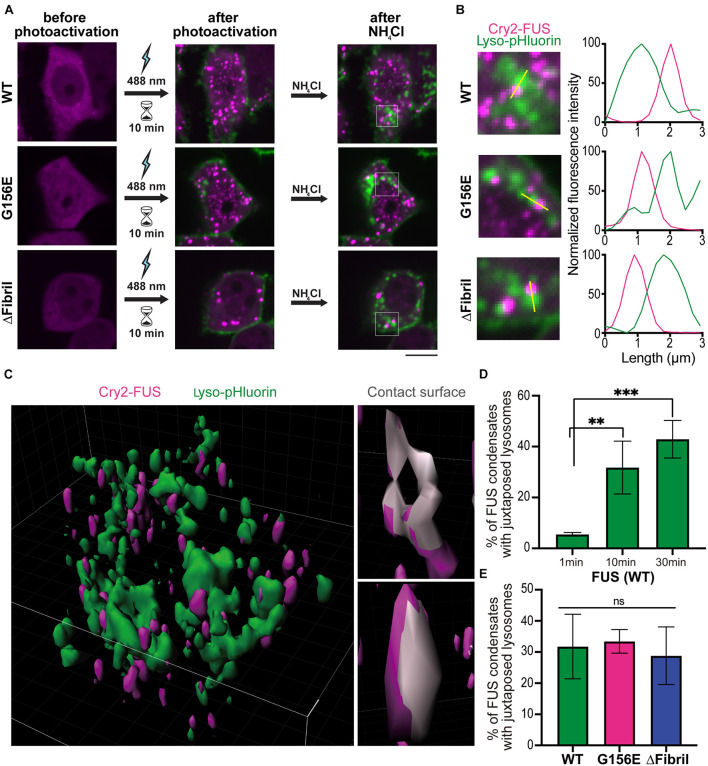
Persistent photoactivation of FUS into solid-like condensates enhances lysosome sequestering. **(A)** Cells co-transfected with lyso-pHluorin and Cry2-FUS (top), Cry2-FUS G156E (middle), and Cry2-FUS ΔFibril (bottom) before (left) and after photoactivation with 488 nm-laser pulses (0.15 mW) for 10 min (middle). Subsequently, ammonium-chloride was added to alkalize the cells and allow for visualization of acidic lysosomes (right). Scale bar, 10 μm. **(B)** Accumulation of lysosomes (lyso-pHluorin) juxtaposed to FUS condensates upon alkalization of cells. Left: magnified regions from images in **(A)**; right: line profiles of the highlighted region indicated with yellow line. **(C)** Exemplary 3D reconstruction volume of lysosomes (lyso-pHluorin, green) and FUS condensates (Cry2-FUS, magenta). Left: Image of an entire cell; right: exemplary FUS condensates with the surface in contact with lysosomes highlighted in gray. **(D)** Colocalization of lysosomes (lyso-pHluorin) with FUS condensates increases with the duration of photoactivation (^∗∗^*P* < 0.01, ^∗∗∗^*P* < 0.001 two-sided, unpaired *t*-test; bars, standard error of the mean). **(E)** Both wild-type and mutated FUS sequences show a similar propensity for lysosome accumulation upon photoactivation.

We further sought to dissect whether lysosome accumulation is specific to a certain condensation state of FUS. To specifically visualize mature, acidic lysosomes, we turned to a pH-sensitive probe specific to lysosome compartments, here referred to as lyso-pHluorin (Rost et al., 2015). Co-expression of lyso-pHluorin with LAMP1-mScarlet followed by the alkalization (Supplementary Figure 5) indicates that only a subset of lysosomes is truly mature and acidic, in line with LAMP1-mScarlet also being expressed on early lysosomes that lack hydrolases (Cheng et al., 2018b). This allowed combining photoactivation with visualization of mature lysosomes. Specifically, we transfected cells with Cry2-FUS constructs and lyso-pHluorin. Upon activation for 10 min using low-intensity laser power (0.15 mW), we generated FUS condensates similarly to Figure 1. We subsequently perfused ammonium-chloride to alkalize the intracellular compartments and visualize the lysosomes (Figure 6A). For wild-type and mutant FUS, we visualized the accumulation of lysosomes juxtaposed to FUS condensates (Figure 6B). This accumulation of lysosomes was absent in the Cry2-only transfected cells that formed no condensates after 10 min of photoactivation (Supplementary Figure 6).

Building on our data that Cry2-FUS activation with low light intensity for extended time produces more solid-like condensates (Figure 3), we decided to systematically analyze whether lysosomes will accumulate more readily around solid-like aggregates or liquid condensates. Specifically, we applied the photoactivation for 1, 10, and 30 min followed by alkalization for visualizing lysosomes. The acquired stacks were processed using the Imaris image analysis suite to determine the fraction of overlapping surfaces between the signal volume arising from FUS condensates and lysosomes (Figure 6C). Notably, the longer the duration of photoactivation – that is, the more fluid condensates are converted to solid-like aggregates – the more lysosomes were accumulated at these sites (Figure 6D). It is important to note that the measured lysosome sequestering around FUS condensates was similar for both wild-type and tested mutants (Figure 6E). Together, these data suggest that lysosomes are responsive to the changes in the material state of the phase and accumulate significantly more around solid-like aggregates than fluid condensates.

## Discussion

ALS genetics implicate RNA-binding proteins such as FUS to play a critical role in disease etiology (Weishaupt et al., 2016). Emerging data suggest that physiological and aberrant biomolecular condensates interact with membranes and membrane-bound organelles (Snead and Gladfelter, 2019). Here, we demonstrate that FUS aggregates but not liquid condensates recruit mature, acidic lysosomes. Specifically, from our data, we can derive three conclusions. First, regardless of whether we use wild-type or mutant FUS, expression levels (i.e., high concentrations) play a dominant role in determining the fraction of cells having soluble or aggregated FUS (Figures 3, 4). Second, chemically induced FUS aggregation sequesters LAMP1-positive structures (Figure 5). Third, coupling Cry2-based manipulation of FUS concentration to pH-sensitive probe for organelle acidity, we dissect that mature, acidic lysosomes accumulate at FUS aggregates but not at liquid-condensates (Figure 6). While we show that disruption of liquid condensation, overexpression, and stress result in FUS aggregation and subsequent recruitment of functional lysosomes, three key questions emerge.

First, does the autophagy-lysosome system act on functionally and compositionally distinct FUS condensates? Physiologically, FUS is important for sensing DNA-damage, regulation of transcription and RNA splicing (Zhou et al., 2014). Upon stress it relocates from the nucleus to the cytosol, where it incorporates into stress granules (Murakami et al., 2015). In neurons, apart from its roles for RNA-processing in the nucleus, FUS regulates the transport of mRNAs into dendrites, a process essential for local protein synthesis during neuronal activity (Aoki et al., 2012). However, during stress, mutated FUS is sequestered within stress granules (Li et al., 2013), which are shown to form through the process of phase separation (Molliex et al., 2015). Indeed, FUS-positive inclusions with other stress granule proteins are present in the brains of patients with ALS (Dormann et al., 2012). The opto-FUS constructs used in this study lack RNA-binding motif (RRM) and nuclear localization sequence (NLS), both of which have been reported to play important roles in modulating the phase states of FUS and localizing the protein together with the associated RNA to subcellular compartments (Dormann et al., 2010; Hofweber et al., 2018). Thus, the subtle difference that G156E mutation causes in condensate formation might be augmented in the case of the full-length FUS protein (Ito et al., 2011). These differential effects of mutations in the IDR region, NLS, or RRM, as well as the interaction with other proteins such as importins/exportins (Baade et al., 2021), certainly warrant being investigated. It also remains to be determined whether different condensation states of FUS interact with a distinct subset of stress granule proteins or specific RNAs (e.g., with specific posttranscriptional modifications, tertiary structures) (Ries et al., 2019; Roden and Gladfelter, 2021).

Second, that aggregation of FUS leads to lysosome sequestering raises a question of what upstream pathways signal this lysosome accumulation. Lysosomal degradation is an important cell mechanism to dismantle unwanted aggregates through the process of autophagy. Autophagy begins by the production of the omegasome, forming the autophagosome membrane, which ultimately fuses with the lysosome. The roles of autophagy in ALS-linked FUS pathology is emerging. For example, a recent study revealed that overriding FUS autoregulation will trigger gain-of-function toxicity via an altered autophagy-lysosome pathway (Ling et al., 2019). Inhibiting lysosomes results in the accumulation of stress granules positive for FUS with autophagosomes, suggest an important role of autophagy axis (Ryu et al., 2014). However, overexpression of Rab1 restored autophagy and the recruitment of mutated FUS to stress granules (Soo et al., 2015). Autophagy is central for clearing aberrant protein aggregates (Buchan et al., 2013), and the dysfunction of autophagosome initiation, maturation, and fusion with the lysosomes is increasingly implicated the pathology of ALS (Burk and Pasterkamp, 2019). Notably, an alternative explanation is the reversible recruitment of lysosomes by FUS-containing RNPs, such as the recently reported lysosome-RNP interaction facilitated by adaptor proteins (e.g., Annexin A11 in Liao et al., 2019). This mechanism is independent of autophagy activation, but a mechanism for RNPs hitchhiking on membrane-bound organelles to promote RNA trafficking and subcellular localization.

Third, how these cellular effects lead to specific vulnerability of motor neurons in ALS. While in the cellular models of ALS, mutated FUS seems to hamper the formation of autophagosome, in ALS patients and model organisms with different protein mutations (e.g., SOD1, TDP-43, FUS) the accumulation of autophagosome might also be a consequence of the activation of macroautophagy, chaperone-mediated autophagy, and microautophagy (Codogno et al., 2012; Nassif et al., 2014; Chen et al., 2015; Xiao et al., 2015; Sellier et al., 2016; Kaushik and Cuervo, 2018). The clearance of aberrant protein aggregates in ALS by lysosome-autophagy axis is also dependent on the surrounding astroglia. Astrocytes expressing ALS-linked mutations in FUS and SOD1 mitigate drug resistance through separate signaling cascades (Qosa et al., 2016). Although these mutation affect distinct pathways, they corroborate downstream in disrupting membrane trafficking (Soo et al., 2015). The combination of light-driven protein condensation with the pH-based detection of lysosomes described here, provides a unique platform to functionally screen for the receptors involved in recognition and clearance of protein aggregates in both neurons and glia.

Together, our work suggests a role for lysosomes in sensing the state of biomolecular condensates and clearing the solid-like inclusions of FUS that are a hallmark of ALS. Disruption of the liquid phase leads to the formation of insoluble aggregates (Shin and Brangwynne, 2017). Interestingly, although different mutations within FUS are suggested to have different pathogenic mechanisms associated with the dynamics of aggregate formation under stress conditions (Patel et al., 2015), impairment of the normal dynamics of phase separation causes lysosome accumulation in all cases (Figure 5). Thus, manipulation of the lysosome dynamics and activity may represent a possible therapeutic strategy for delaying disease progression in ALS cases associated with FUS mutations (Marrone et al., 2019) and related neurodegenerative diseases where proteins able to phase separate could form aberrant insoluble aggregates (Wallings et al., 2019).

## Data Availability Statement

The original contributions presented in the study are included in the article/Supplementary Material, further inquiries can be directed to the corresponding author.

## Author Contributions

FT, CH, HW, and DM contributed to the conception and design of the experiments. FT, CH, HW, RS, BR, BRR, HBS, and DM performed data acquisition and analysis. FT, CH, BRR, DS, and HBS were involved in data interpretation and wrote the manuscript. All authors read and approved the final manuscript for submission.

## Conflict of Interest

The authors declare that the research was conducted in the absence of any commercial or financial relationships that could be construed as a potential conflict of interest.

## Publisher’s Note

All claims expressed in this article are solely those of the authors and do not necessarily represent those of their affiliated organizations, or those of the publisher, the editors and the reviewers. Any product that may be evaluated in this article, or claim that may be made by its manufacturer, is not guaranteed or endorsed by the publisher.

## Supplementary Material

Supplementary Figure 1 **(A)** Diameter of condensates after photoactivation (1 min at 0.02 mW 488 nm-laser power at the output) of Cry2-mCherry-FUS for wild-type sequence (green), G156E mutation (magenta), and ΔFibril (blue). **(B)** Percentage of activated cells expressing Cry2-mCherry-FUS wild-type sequence (green), G156E mutation (magenta), and ΔFibril (blue) under activation conditions same as in **(A)**. ^∗∗^*P* < 0.01, two-sided, unpaired *t*-test; each experiment is done in three different rounds of transfections.

Supplementary Figure 2 Photoactivation (1 min) of FUS condensation. Images (left) and diameter of droplets (right) for wild-type sequence **(A,B)**, G156E mutation **(C,D)**, and ΔFibril **(E,F)** at 1, 5, and 10% of 488- laser power (corresponding to 0.01, 0.04, and 0.15 mW, respectively). Scale bar, 10 μm.

Supplementary Figure 3 **(A)** Expression of three FUS-EGFP variants analyzed by immunoblotting. **(B)** Frequency of condensates with a specific circularity (aspect ratio) in cells transfected with 1 μg of FUS-EGFP sequence for wild-type (green), G156 E (magenta) and ΔFibril (blue) protein.

Supplementary Figure 4 Increasing concentration of cytosolic FUS leads to aggregation for both wild-type and mutated proteins. **(A)** Images (z-projections) of HEK cells expressing wild-type FUS (left), FUS G156E (middle) and ΔFibril (right) before (top) and upon addition of 3% 1,6-hexanediol (bottom). Each condition was transfected with 1.5 μg of corresponding plasmid. **(B)** The percentage of cells that contain soluble FUS decreases with increasing amounts of transfected FUS-EGFP construct. **(C)** The percentage of cells that contain either soluble or aggregated wild-type FUS (green), FUS G156E (magenta), and FUS ΔFibril (blue) when transfected with 0.3 μg of the corresponding construct. ^∗∗^*P* < 0.01, two-sided, unpaired *t*-test; bars. Bars represent average values with standard deviation; each experiment is done in three different rounds of transfections with at least 200 analyzed cells for each condition. Scale bar, 10 μm.

Supplementary Figure 5 Lyso-pHluorin labels a subset of LAMP1-positive structures. Images of cells co-transfected with LAMP1-mScarlet (marker of late endosomes and lysosomes) and lyso-pHluorin (marker of acidic lysosomes) before (top) and after (bottom) the alkalization with ammonium-chloride. Scale bar, 10 μm.

Supplementary Figure 6 Cells co-transfected with Cry2-mCherry and lyso-pHluorin form no condensates upon photoactivation of Cry2-mCherry with 488 nm-laser (0.15 mW) for 10 min.

Supplementary Table 1 DNA sequence of the Cry2-mCherry-MCS expression cassette with restriction sites for subsequent cloning of FUS constructs: XhoI and KpnI.

Supplementary Table 2 Primer sequences for Cry2-mCherry-FUS (1-215) subcloning (XhoI and KpnI) and primers for mutants generated by QC mutagenesis PCR.
