# Supplementary figures and images for: Aberrant Phase Separation of FUS Leads to Lysosome Sequestering and Acidification
Source: Front Cell Dev Biol. 2021 Oct 22;9:716919. doi: 10.3389/fcell.2021.716919 (PMC8569517; doi:10.3389/fcell.2021.716919)

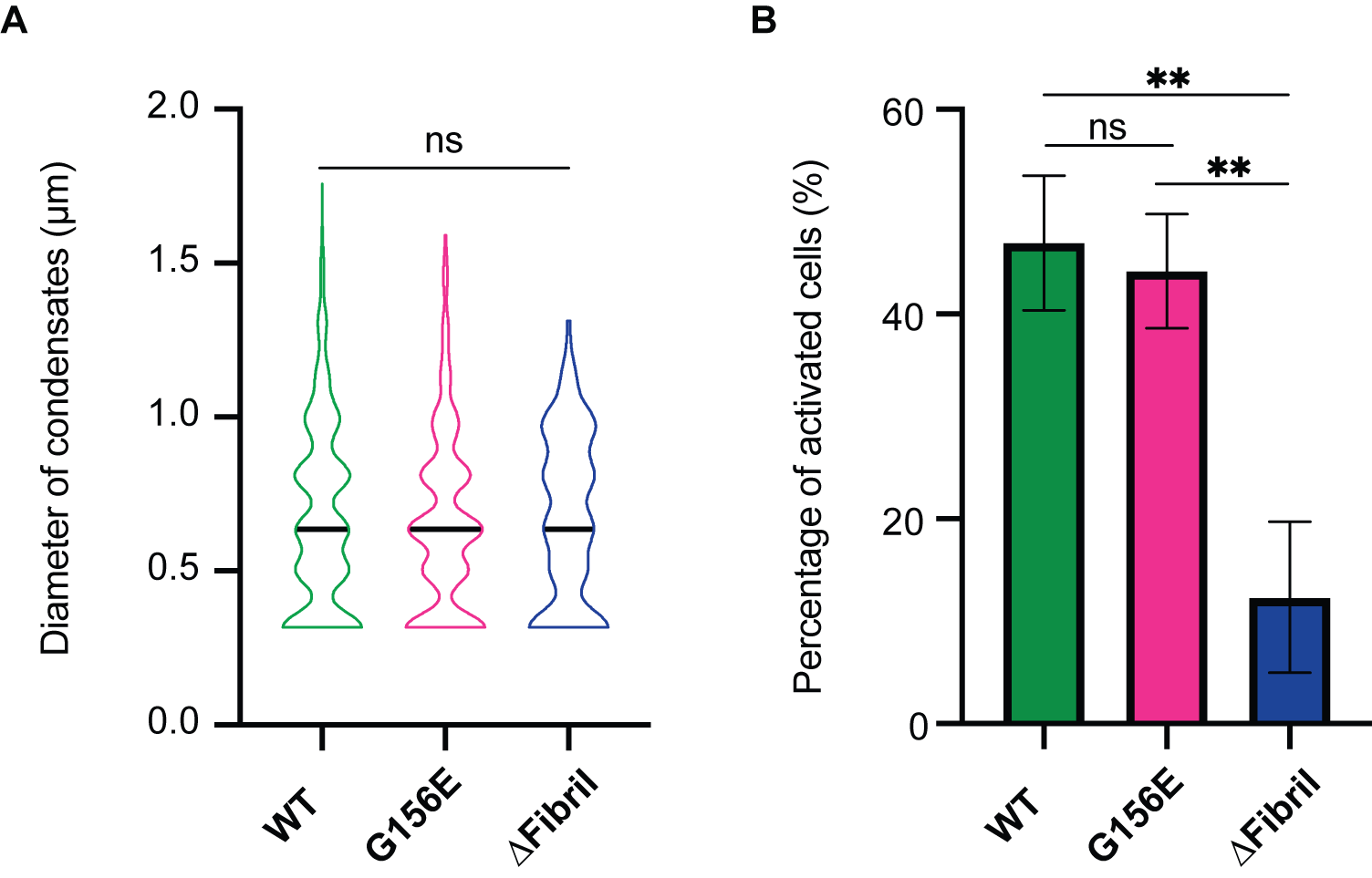

Supplement: Supplementary Figure 1 — (A) Diameter of condensates after photoactivation (1 min at 0.02 mW 488 nm-laser power at the output) of Cry2-mCherry-FUS for wild-type sequence (green), G156E mutation (magenta), and ΔFibril (blue). (B) Percentage of activated cells expressing Cry2-mCherry-FUS wild-type sequence (green), G156E mutation (magenta), and ΔFibril (blue) under activation conditions same as in (A). ∗∗P < 0.01, two-sided, unpaired t-test; each experiment is done in three different rounds of transfections. [file Image_1.TIF]

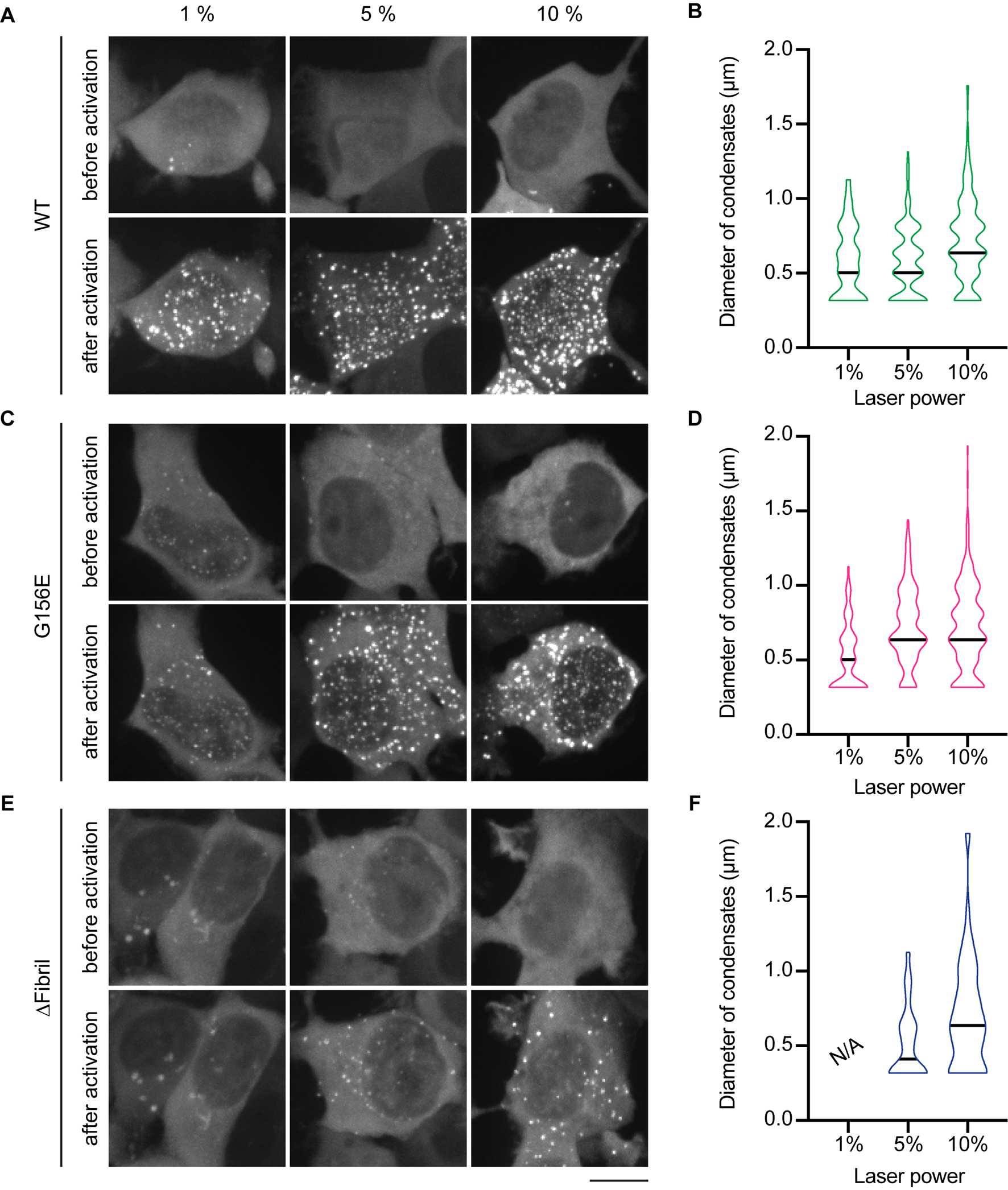

Supplement: Supplementary Figure 2 — Photoactivation (1 min) of FUS condensation. Images (left) and diameter of droplets (right) for wild-type sequence (A,B), G156E mutation (C,D), and ΔFibril (E,F) at 1, 5, and 10% of 488- laser power (corresponding to 0.01, 0.04, and 0.15 mW, respectively). Scale bar, 10 μm. [file Image_2.TIF]

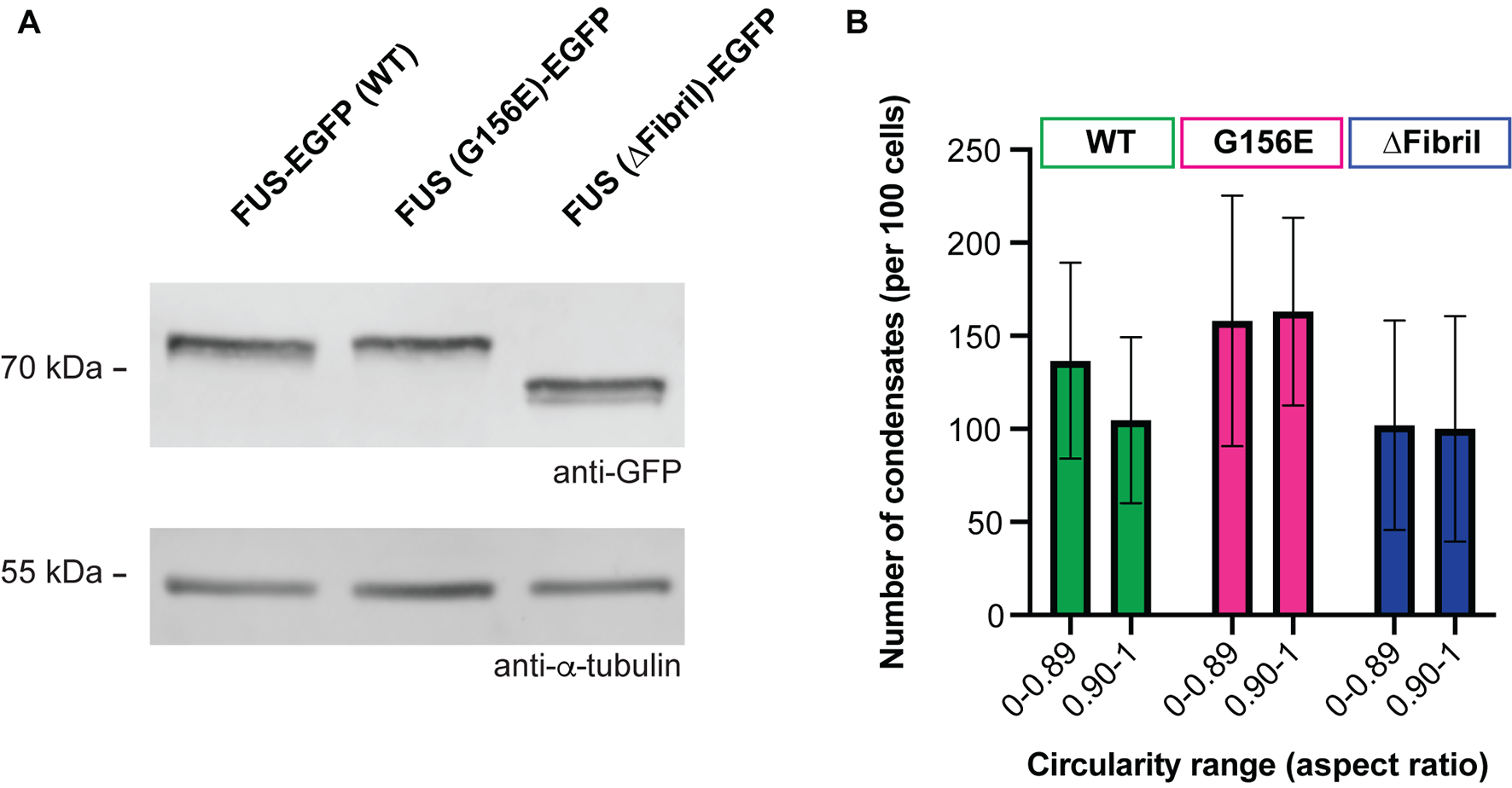

Supplement: Supplementary Figure 3 — (A) Expression of three FUS-EGFP variants analyzed by immunoblotting. (B) Frequency of condensates with a specific circularity (aspect ratio) in cells transfected with 1 μg of FUS-EGFP sequence for wild-type (green), G156 E (magenta) and ΔFibril (blue) protein. [file Image_3.TIF]

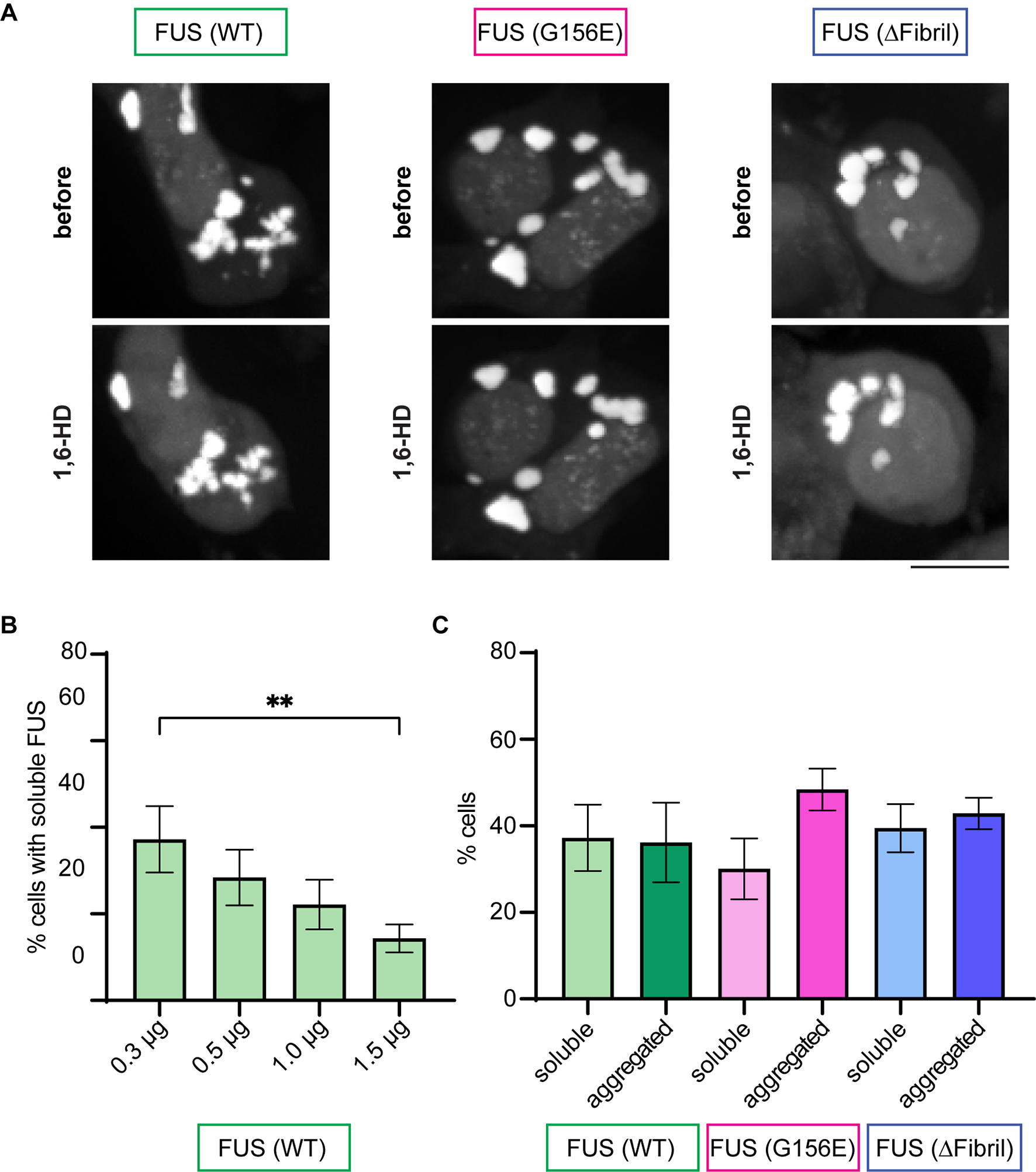

Supplement: Supplementary Figure 4 — Increasing concentration of cytosolic FUS leads to aggregation for both wild-type and mutated proteins. (A) Images (z-projections) of HEK cells expressing wild-type FUS (left), FUS G156E (middle) and ΔFibril (right) before (top) and upon addition of 3% 1,6-hexanediol (bottom). Each condition was transfected with 1.5 μg of corresponding plasmid. (B) The percentage of cells that contain soluble FUS decreases with increasing amounts of transfected FUS-EGFP construct. (C) The percentage of cells that contain either soluble or aggregated wild-type FUS (green), FUS G156E (magenta), and FUS ΔFibril (blue) when transfected with 0.3 μg of the corresponding construct. ∗∗P < 0.01, two-sided, unpaired t-test; bars. Bars represent average values with standard deviation; each experiment is done in three different rounds of transfections with at least 200 analyzed cells for each condition. Scale bar, 10 μm. [file Image_4.TIF]

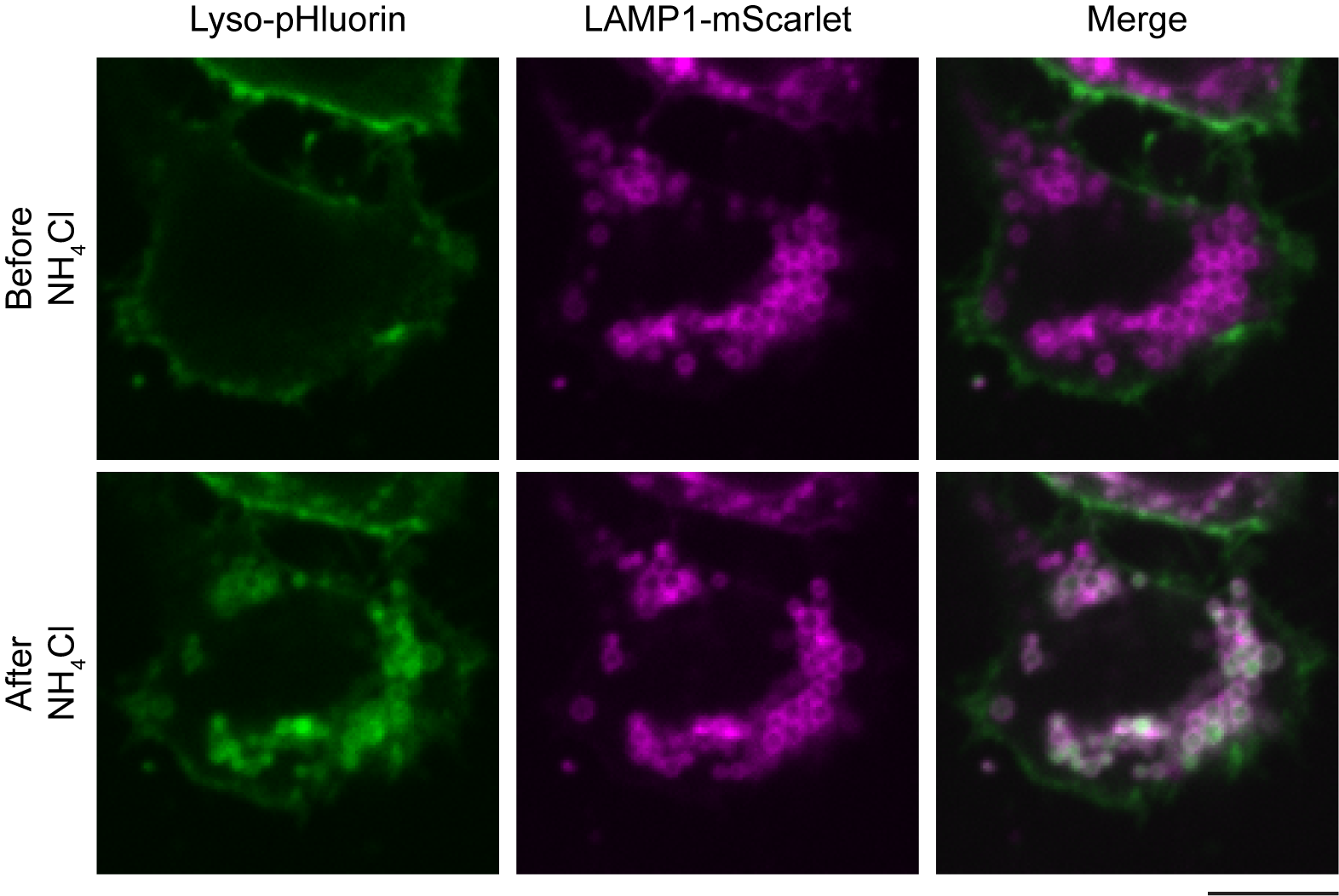

Supplement: Supplementary Figure 5 — Lyso-pHluorin labels a subset of LAMP1-positive structures. Images of cells co-transfected with LAMP1-mScarlet (marker of late endosomes and lysosomes) and lyso-pHluorin (marker of acidic lysosomes) before (top) and after (bottom) the alkalization with ammonium-chloride. Scale bar, 10 μm. [file Image_5.TIF]

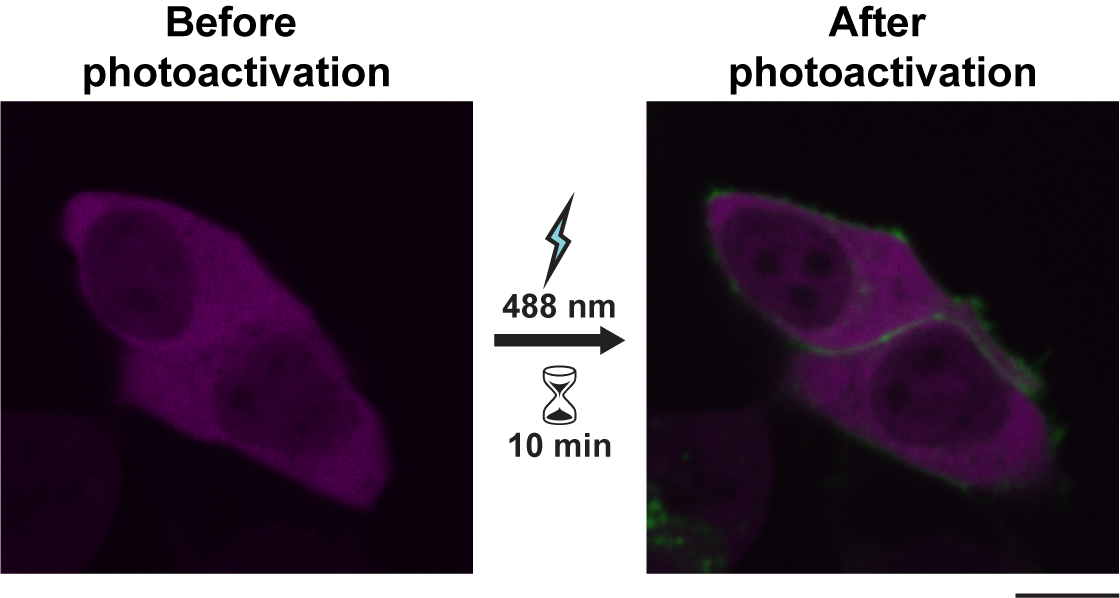

Supplement: Supplementary Figure 6 — Cells co-transfected with Cry2-mCherry and lyso-pHluorin form no condensates upon photoactivation of Cry2-mCherry with 488 nm-laser (0.15 mW) for 10 min. [file Image_6.TIF]
