# Supplementary material for: Aberrant Phase Separation of FUS Leads to Lysosome Sequestering and Acidification
Source: Front Cell Dev Biol. 2021 Oct 22;9:716919. doi: 10.3389/fcell.2021.716919 (PMC8569517; doi:10.3389/fcell.2021.716919)
Supplement: Supplementary Table 1 — DNA sequence of the Cry2-mCherry-MCS expression cassette with restriction sites for subsequent cloning of FUS constructs: XhoI and KpnI. [file Table_1.pdf]

Supplementary Table 1. DNA sequence of the Cry2-mCherry-MCS expression cassette with restriction sites for subsequent cloning of FUS constructs: XhoI and KpnI.

Expression cassette: cmv-Cry2-**mCherry**-MCS-*SV4*

cgccccattgacgcaaatgggcggtaggcgtgtacggtgggaggtctatataagcagagctggtttagtgaaccgtcagatcCGCTAGCGCTAC
CGGTCGCCACCATGAAGATGGACAAAAAGACTATAGTTTGGTTTAGAAGAGACCTAAGGATTG
AGGATAATCCTGCATTAGCAGCAGCTGCTCACGAAGGATCTGTTTTTCCTGTCTTCATTTGGTG
TCCTGAAGAAGAAGGACAGTTTTATCCTGGAAGAGCTTCAAGATGGTGGATGAAACAATCACT
TGCTCACTTATCTCAATCCTTGAAGGCTCTTGGATCTGACCTCACTTTAATCAAAACCCACAAC
ACGATTTCAGCGATCTTGGATTGTATCCGCGTTACCGGTGCTACAAAAGTCGTCTTTAACCACC
TCTATGATCCTGTTTCGTTAGTTCGGGACCATACCGTAAAGGAGAAGCTGGTGGAACGTGGGA
TCTCTGTGCAAAGCTACAATGGAGATCTATTGTATGAACCGTGGGAGATATACTGCGAAAAGG
GCAAACCTTTTACGAGTTTCAATTCTTACTGGAAGAAATGCTTAGATATGTCGATTGAATCCGT
TATGCTTCCTCCTCCTTGGCGGTTGATGCCAATAACTGCAGCGGCTGAAGCGATTTGGGCGTGT
TCGATTGAAGAACTAGGGCTGGAGAATGAGGCCGAGAAACCGAGCAATGCGTTGTTAACTAG
AGCTTGGTCTCCAGGATGGAGCAATGCTGATAAGTTACTAAATGAGTTCATCGAGAAGCAGTT
GATAGATTATGCAAAGAACAGCAAGAAAGTTGTTGGGAATTCTACTTCACTACTTTCTCCGTAT
CTCCATTTCGGGGAAATAAGCGTCAGACACGTTTTCCAGTGTGCCCGGATGAAACAAATTATA
TGGGCAAGAGATAAGAACAGTGAAGGAGAAGAAAGTGCAGATCTTTTTCTTAGGGGAATCGG
TTTAAGAGAGTATTCTCGGTATATATGTTTCAACTTCCCGTTTACTCACGAGCAATCGTTGTTG
AGTCATCTTCGGTTTTTCCCTTGGGATGCTGATGTTGATAAGTTCAAGGCCTGGAGACAAGGCA
GGACCGGTTATCCGTTGGTGGATGCCGGAATGAGAGAGCTTTGGGCTACCGGATGGATGCATA
ACAGAATAAGAGTGATTGTTTCAAGCTTTGCTGTGAAGTTTCTTCTCCTTCCATGGAAATGGGG
AATGAAGTATTTCTGGGATACACTTTTGGATGCTGATTTGGAATGTGACATCCTTGGCTGGCAG
TATATCTCTGGGAGTATCCCCGATGGCCACGAGCTTGATCGCTTGGACAATCCCGCGTTACAAG
GCGCCAAATATGACCCAGAAGGTGAGTACATAAGGCAATGGCTTCCCGAGCTTGCGAGATTGC
CAACTGAATGGATCCATCATCCATGGGACGCTCCTTTAACCGTACTCAAAGCTTCTGGTGTGGA
ACTCGGAACAAACTATGCGAAACCCATTGTAGACATCGACACAGCTCGTGAGCTACTAGCTAA
AGCTATTTCAAGAACCCGTGAAGCACAGATCATGATCGGAGCAGCAGCCCGGGATCCACCGGT
CGCCACC**ATGGTGAGCAAGGGCGAGGAGGATAACATGGCCATCATCAAGGAGTTCATGC**
GCTTCAAGGTGCACATGGAGGGCTCCGTGAACGGCCACGAGTTCGAGATCGAGGGCGAG
GGCGAGGGCCGCCCCTACGAGGGCACCCAGACCGCCAAGCTGAAGGTGACCAAGGGTG
GCCCCCTGCCCTTCGCCTGGGACATCCTGTCCCCTCAGTTCATGTACGGCTCCAAGGCCT
ACGTGAAGCACCCCGCCGACATCCCCGACTACTTGAAGCTGTCCTTCCCCGAGGGCTTCA
AGTGGGAGCGCGTGATGAACTTCGAGGACGGCGGCGTGGTGACCGTGACCCAGGACTCC
TCCCTGCAGGACGGCGAGTTCATCTACAAGGTGAAGCTGCGCGGCACCAACTTCCCCTC
CGACGGCCCCGTAATGCAGAAGAAGACCATGGGCTGGGAGGCCTCCTCCGAGCGGATGT
ACCCCGAGGACGGCGCCCTGAAGGGCGAGATCAAGCAGAGGCTGAAGCTGAAGGACGG
CGGCCACTACGACGCTGAGGTCAAGACCACCTACAAGGCCAAGAAGCCCGTGCAGCTGC
CCGGCGCCTACAACGTCAACATCAAGTTGGACATCACCTCCCACAACGAGGACTACACCA
TCGTGGAACAGTACGAACGCGCCGAGGGCCGCCACTCCACCGGCGGCATGGACGAGCTG
TACAAGTCCGGACTCGGCGGCCACCGCTGGATCCTGGAGGTGCTGTTCCAGGGCCCCAGATCT
CGAGCTCAAGCTTCGAATTCTGCAGTCGACGGTACCGCGGGCCCGGGATCCACCGGATCTAGA
TAAC*TGATCATAATCAGCCATACCACATTTGTAGAGGTTTTACTTGCTTTAAAAAACCTCCCACACCTC*
CCCCTGAACCTGAAACATAAAATGAATGCAATTGTTGTTGTTAACTTGTTTATTGCAGCTTATAATGGTT
ACAAATAAAGCAATAGCATCACAAATTTCACAAATAAAGCATTTTTTTCACTGCATTCTAGTTGTGGTTT
GTCCAAACTCATCAATGTATCTTAACGCGTAAATTGTAAG
