# Supplementary material for: Aberrant Phase Separation of FUS Leads to Lysosome Sequestering and Acidification
Source: Front Cell Dev Biol. 2021 Oct 22;9:716919. doi: 10.3389/fcell.2021.716919 (PMC8569517; doi:10.3389/fcell.2021.716919)
Supplement: Supplementary Table 2 — Primer sequences for Cry2-mCherry-FUS (1-215) subcloning (XhoI and KpnI) and primers for mutants generated by QC mutagenesis PCR. [file Table_2.pdf]

Supplementary Table 2. Primer sequences for Cry2-mCherry-FUS (1-215) subcloning (XhoI and KpnI) and primers for mutants generated by QC mutagenesis PCR.

**Cry2-mCherry-FUS$_{WT}$**	**FW:** ATCACTCGAGCTATGGCCTCAAACGATTATACC<br>**RV:** ATCAGGTACCTCATTAACCTCCACGGTCCTGCTGTC
**Cry2-mCherry-FUS$_{G156E}$**	**FW:** TCAGGGCTATGAGCAGCAGAACCAGTACAACAGC<br>**RV:** GGTTCTGCTGCTCATAGCCCTGAGGGGGATTATAG
**Cry2-mCherry-FUS$_{\Delta Fibril}$**	**FW:** CAGCAGAGTTACTCCTATCCTGGCTATGGCCAG<br>**RV:** GCCAGGATAGGAGTAACTCTGCTGTCCGTAGG
